# Editorial: Sustainable and intelligent plant health management in Asia (2022)

**DOI:** 10.3389/fpls.2023.1244869

**Published:** 2023-08-14

**Authors:** Kuo-Hui Yeh, Ilias Travlos, Ahmad Nawaz, Shih-Chieh Chang, Han-Chieh Chao

**Affiliations:** ^1^ Department of Information Management, National Dong Hwa University, Hualien, Taiwan; ^2^ Department of Computer Science and Engineering, National Sun Yat-sen University, Kaohsiung, Taiwan; ^3^ Faculty of Crop Science, Agricultural University of Athens, Athens, Greece; ^4^ Department of Plant Pathology, University of Agriculture, Faisalabad, Pakistan; ^5^ Department of Natural Resources and Environmental Studies, National Dong Hwa University, Hualien, Taiwan; ^6^ Department of Electrical Engineering, National Dong Hwa University, Hualien, Taiwan; ^7^ Institute of Computer Science and Innovation, UCSI University, Kuala Lumpur, Malaysia

**Keywords:** smart phytoprotection, plant health management, intelligent control and analytic technology, sustainable plant protection, Asia

Phytoprotection encompasses various aspects of safeguarding plants, including plant pathology, etiology, molecular and population biology, breeding biotechnology, environmental stress management, and pest control. In recent times, the academic community has increasingly focused on integrating the traditionally biology-oriented field of plant protection with emerging intelligent technologies. This convergence has given rise to a groundbreaking concept known as smart phytoprotection, which leverages cutting-edge intelligent control and analytic technologies to explore innovative research ideas and solutions for sustainable plant protection and development. Key areas of interest within smart phytoprotection include precise ecological modeling, cost-effective and reliable edge equipment for ecological control, lightweight and intelligent computing for accurate plant protection, and open data for collaborative intelligence sharing. Broadly speaking, smart phytoprotection operates at the intersection of multidisciplinary intelligent control and analytic technologies, such as the Internet of Things (IoT), satellite remote sensing, 5G/6G networking, edge computing, big data analytics, machine learning, blockchain, robotics, cognitive computing, and artificial intelligence. These transformative technologies empower continuous advancements and achievements in plant/crop protection. However, as the interest in smart phytoprotection grows, it becomes crucial to address the challenges associated with integrating intelligent control and analytic technologies into the field of plant protection.

The aim of this Research Topic is to consolidate the latest research endeavors focused on exploring emerging intelligent control and analytic technologies for smart phytoprotection and plant health management. Following a thorough peer-review process, we have selected four outstanding research contributions for publication within this Research Topic:

## 1 “Intelligent image analysis recognizes important orchid viral diseases”

This study proposes an intelligent image analysis approach for the accurate recognition of important viral diseases in orchids. The research demonstrates the efficacy of this method in early detection and diagnosis, aiding in the effective management and protection of orchid crops.

## 2 “Assessing farmland suitability for agricultural machinery in land consolidation schemes in hilly terrain in China: a machine learning approach”

This research applies a machine learning approach to evaluate farmland suitability for agricultural machinery in land consolidation projects conducted in hilly terrains in China. The study highlights the potential of machine learning models in optimizing machinery allocation and enhancing agricultural productivity in challenging topographic conditions.

## 3 “Detecting strawberry diseases and pest infections in the very early stage with an ensemble deep-learning model”

This study proposes an ensemble deep-learning model for the early detection of diseases and pest infections in strawberry plants. By analyzing various visual cues, the research demonstrates the effectiveness of the model in identifying and diagnosing plant health issues at their incipient stages, enabling timely intervention and improved crop protection.

## 4 “Toxicity and synergistic activity of compounds from essential oils and their effect on detoxification enzymes against Planococcus lilacinus”

This research investigates the toxicity and synergistic activity of compounds derived from essential oils against Planococcus lilacinus, a common agricultural pest. The study assesses the impact of these compounds on detoxification enzymes, providing valuable insights into their potential as eco-friendly alternatives for pest management.

These selected research articles offer significant contributions to the field of smart phytoprotection and plant health management, showcasing innovative applications of intelligent control and analytic technologies.

In the paper titled “*Intelligent Image Analysis Recognizes Important Orchid Viral Diseases*” by Tsai et al., the authors present a system for detecting orchid viral diseases by image analysis. The system comprises five components: (a) preprocessing the image by applying color space transformation and gamma correction, (b) employing a U-net model to detect leaves, (c) removing non-leaf fragments using connected component labeling, (d) acquiring leaf texture features, and (e) identifying diseases using a two-stage model that integrates a random forest model and an inception network model. The proposed system achieves high accuracy, with 0.9707 for orchid leaf image segmentation and 0.9180 for disease identification. Notably, the system significantly improves the accuracy of virus identification for cymbidium mosaic virus (CymMV) and odontoglossum ringspot virus (ORSV) compared to human-eye-based identification. The accuracy rates for the proposed two-stage model are 0.842, while human-eye-based identification achieves an accuracy rate of 0.667.

In the paper titled “*Assessing Farmland Suitability for Agricultural Machinery in Land Consolidation Schemes in Hilly Terrain in China: A Machine Learning Approach*” by Yang et al., the authors employ a machine learning approach to assess the suitability of farmland for agricultural machinery in land consolidation schemes in hilly terrains in China. The study considers factors such as natural resource endowment, accessibility of agricultural machinery, socioeconomic level, and ecological limitations. The authors divide the farmlands into four categories based on suitability and potential improvement in farmland productivity: the priority consolidation zone, the moderate consolidation zone, the comprehensive consolidation zone, and the reserve consolidation zone. The research findings indicate that approximately 76.41% of the farmland is either basically or moderately suitable for Farmland Consolidation suitable for Agricultural Machinery (FCAM). Implementing FCAM could increase the potential productivity of farmland by 720.8 kg/ha. The proposed zone suggestion serves as a useful basis for determining implementation sequences and differentiation strategies for FCAM schemes.

The third paper titled “*Detecting Strawberry Diseases and Pest Infections in the Very Early Stage with an Ensemble Deep-Learning Model*” is proposed by (Lee et al.). The authors develop an automatic data acquisition system to collect a large amount of plant image data, specifically 13,393 images with nearly 120,000 objects, in order to detect early signs of plant diseases and pests and minimize potential damages. They establish an object detection model using the YOLO v5 algorithm and build an ensemble detection model with nine independent models to improve detection performance. The ensemble system model achieves a decent detection performance with an average AUPRC (Area Under Precision-Recall Curve) rate of 0.819. The authors incorporate their proposed imaging system and deep learning-based detection module into a periodic and automatic monitoring system for strawberry plants. Additionally, the system includes a notification functionality to automatically inform users of early symptoms of diseases or pests.

Lastly, in the paper titled “*Toxicity and Synergistic Activity of Compounds from Essential Oils and their Effect on Detoxification Enzymes against Planococcus lilacinus*” by Arokiyaraj et al. the authors investigate the insecticidal activities of pure compounds from essential oils against P. lilacinus. The experimental results show that the pure compounds L-limonene, β-myrcene, and ocimene exhibit toxicity, each with an LD50 of 0.37 µg/insect after 96 hours. The binary mixtures of geraniol + L-menthol and L-limonene + geraniol exhibit synergistic effects, each with an LD50 of 0.03 µg/insect after 96 hours. At higher concentrations of 5,000 ppm, the monoterpenes ocimene and β-myrcene substantially inhibit the activities of detoxification enzymes AChE (0.93 and 0.78 mU/mg, respectively) and GST (2.19 and 7.29 nmol/min/ml, respectively) in P. lilacinus after 48 hours. SEM analysis reveals significant anomalies in the morphology of the abdominal cuticle, setae, and thoracic leg after a 96-hour treatment with ocimene at 1,250 ppm against P. lilacinus. The tested pure compounds and their combinations show promise for the control of mealybugs.

## Author contributions

The topic editors, K-HY, IT, AN, S-CC, and H-CC, have taken on the responsibilities of organizing, promoting, and processing the manuscripts for this Research Topic. They have played a crucial role in coordinating the submission and review process, ensuring the smooth progression of the topic. Their efforts in overseeing the publication process have been instrumental in bringing together a collection of high-quality research articles in this field. All authors contributed to the article and approved the submitted version.

